# Association between C-reactive protein-triglyceride glucose index and NAFLD in US adults: A nationally cross-sectional study using NHANES 2017-2020 data

**DOI:** 10.1097/MD.0000000000045777

**Published:** 2025-11-07

**Authors:** Yuanyuan Chen, Jinmin Cao, Huihui Chen, Bing Yang, Jun Chen, Chuantie Chen

**Affiliations:** a Department of Liver Diseases, Shenzhen Third People’s Hospital, The Second Affiliated Hospital of Southern University of Science and Technology, National Clinical Research Center for Infectious Diseases, Shenzhen, Guangdong, China; b Department of Dermatology, Hunan Aerospace Hospital, Changsha, Hunan, China; c Guangzhou University of Traditional Chinese Medicine, Guangzhou, Guangdong, China; d Department of Gastroenterology, Longgang Central Hospital of Shenzhen, Shenzhen, Guangdong, China.

**Keywords:** controlled attenuation parameter, C-reactive protein-triglyceride glucose index, hepatic steatosis, NAFLD, NHANES

## Abstract

The C-reactive protein-triglyceride glucose index (CTI) is a novel biomarker for assessing insulin resistance and the severity of inflammation. However, its association with nonalcoholic fatty liver disease in United States adults has not been well established. Using data from the 2017 to 2020 National Health and Nutrition Examination Survey, this cross-sectional study applied multivariate linear regression models to investigate the linear relationship between CTI and controlled attenuation parameters (CAP). In addition, we performed stratified and interaction analyses to evaluate the stability of the relationships within different subgroups. The study enrolled 3248 participants, comprising 1474 males (45.4%) and 1774 females (54.6%) with a median age of 49 years. All 3 models demonstrated a positive association between CTI and CAP: Model 1 (beta [β] = 33.79; 95% confidence interval [CI]: 31.8–35.78), Model 2 (β = 31.80; 95% CI: 29.75–33.86), and Model 3, a fully adjusted model, (β = 13.85; 95% CI: 11.51–16.2). The CTI was stratified into quartiles, and a trend test indicated a linear relationship between the CTI and CAP across all 3 models (*P* for trend < .05). Similarly, smooth curve analysis indicated a linear relationship (*P* for nonlinearity = .178). The subgroup analyses also demonstrated a positive association in each subgroup. The CTI is positively associated with CAP in adults in the United States, suggesting its potential as a reliable indicator for nonalcoholic fatty liver disease monitoring.

## 1. Introduction

Nonalcoholic fatty liver disease (NAFLD), which affects up to 30.2% of the world’s population, is the most common chronic liver disease.^[1]^ The disease can progress from simple hepatic steatosis to nonalcoholic steatohepatitis, followed by liver fibrosis, cirrhosis, and eventually hepatocellular carcinoma.^[2,3]^ Excessive fat accumulation in the liver leads to hepatocyte dysfunction, pro-inflammatory immune response activation, and fibrogenesis. It also leads to various extrahepatic metabolic disorders, such as cardiovascular events and type 2 diabetes mellitus.^[4]^ Furthermore, Powell et al highlighted the link between hepatic steatosis, fibrosis, and increased all-cause mortality.^[5]^ Over the next decade, NAFLD is expected to become the primary indication for liver transplantation in numerous countries.^[6]^ In 2020, an international expert consensus proposed renaming NAFLD to metabolic dysfunction-associated fatty liver disease to better reflect its metabolic pathogenesis.^[7]^ Given the substantial overlap between metabolic dysfunction-associated fatty liver disease and NAFLD populations, and to maintain compatibility with the longitudinal data in this study (2017–2020) as well as consistency with the original diagnostic criteria, this paper continues to use the term NAFLD.

The accurate determination of the extent of hepatic steatosis is crucial for the evaluation and clinical prognosis of patients with NAFLD.^[8]^ Although liver biopsy remains the gold standard for assessing hepatic steatosis and fibrosis, vibration-controlled transient elastography (VCTE) is increasingly being adopted as a noninvasive diagnostic tool in clinical practice. VCTE evaluates the severity of liver steatosis and fibrosis by measuring 2 key parameters: the controlled attenuation parameter (CAP) for steatosis and liver stiffness measurement (LSM) for fibrosis. Studies have demonstrated the high diagnostic accuracy of CAP in quantifying hepatic steatosis.^[8,9]^ Compared with invasive biopsy, VCTE provides a reliable, noninvasive alternative for liver disease assessment.^[10]^

Evidence suggests that inflammation and insulin resistance (IR) play crucial roles in the development and progression of NAFLD. Patients with NAFLD exhibit systemic low-grade inflammation, and a meta-analysis by Duan et al indicated that elevated inflammatory markers such as C-reactive protein (CRP) are significantly associated with NAFLD development.^[11]^ CRP is a classic nonspecific acute-phase protein produced by the liver and an indicator of systemic inflammation.^[12]^ IR is the core element of metabolic syndrome. NAFLD is characterized by an impaired response to insulin.^[13]^ IR can cause metabolic imbalance, increase oxidative stress, and amplify the inflammatory response.^[14,15]^ Although the hyperinsulinemic-euglycemic clamp test is the gold standard for assessing IR, the triglyceride glucose index has shown superior sensitivity and specificity for its identification.^[16]^ A pro-inflammatory state has been shown to induce IR. In this state, the anti-inflammatory effects of insulin are impaired, and free fatty acid concentrations increase, further exacerbating the inflammatory response.^[17]^ The reciprocal relationship between inflammation and IR intensifies both conditions, ultimately resulting in NAFLD symptoms.^[18]^

The CRP-triglyceride glucose comprehensive index (CTI) was initially developed by Ruan et al in 2022^[19]^ as a novel composite biomarker. It serves as an integrated indicator that simultaneously reflects both the inflammatory state and IR status. Research findings have demonstrated that the CTI accurately evaluates survival of patients with cancer, supporting its use for mortality stratification.^[20]^ The innovative CTI, an accessible, cost-effective, and quick index based on biochemical tests, has an underexplored correlation with NAFLD. This study aimed to investigate the relationship between the CTI and NAFLD by considering the existing limitations. To this end, a large cohort from the National Health and Nutrition Examination Survey (NHANES) was utilized in order to provide robust evidence for this association.

## 2. Materials and methods

### 2.1. Study population

**Figure 1.**
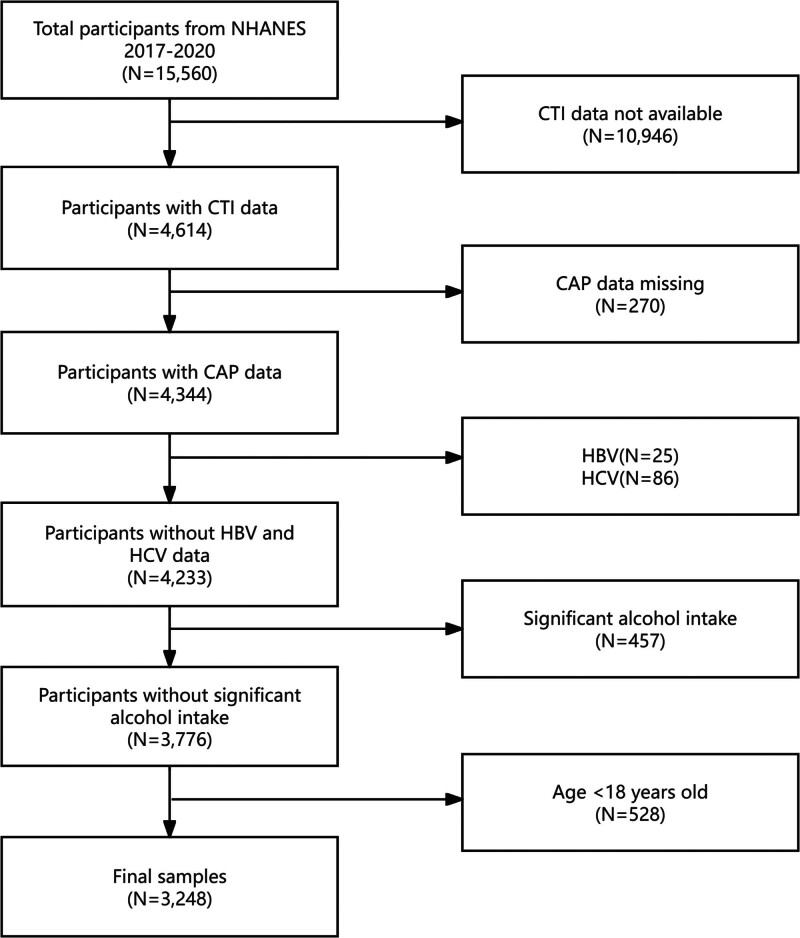
Flowchart of the study participants. CAP = controlled attenuation parameter, CTI = C-reactive protein-triglyceride glucose index, NHANES = National Health and Nutrition Examination Survey.

The Centers for Disease Control and Prevention conducts the NHANES, which is designed to represent the national population. The study procedure received approval from the National Center for Health Statistics Research Ethics Review Board. The participants provided their written informed consent to participate in this study. And secondary analysis does not require additional institutional review committee approval. Our study was conducted using the pre-pandemic NHANES data from 2017 to 2020. At baseline, there were 15,560 participants in the cohort, which was screened for the current study. There were 10,946 individuals with missing CTI data, 270 with incomplete CAP data, 25 who were hepatitis B surface antigen-positive, and 86 with positive results for hepatitis C antibodies or RNA. A further 457 participants with a significant alcohol consumption (defined as having 4/5 or more drinks per day) and 528 participants aged <18 years were excluded. Following the screening process, 3248 participants were deemed eligible for inclusion. Figure 1 illustrates a flowchart of the sample selection process.

### 2.2. Exposure and outcome definition

The study’s exposure variable, CTI, was derived from the biochemical data of blood samples, including high-sensitivity CRP (hs-CRP), triglycerides, and fasting blood glucose (FBG), with triglycerides and fasting glucose indicating IR. Biochemical samples were collected in the morning after an 8-hour fast and sent to an National Center for Health Statistics-accredited laboratory. Hs-CRP was quantified using a 2-reagent immunoturbidimetric assay on a Roche Cobas 6000 clinical chemistry analyzer, while FBG was measured using the oxygen rate method on a Beckman DxC800. The CTI was determined using the equation CTI = 0.412 × Ln[hs-CRP (mg/L)] + Ln[triglycerides (mg/dL) × FBG (mg/dL)/2].

The outcome variable in this study was CAP, which was measured using VCTE, and indicated the degree of hepatic steatosis. Higher CAP values correspond to more severe hepatic steatosis. This study identified a median CAP of 274 dB/m as the optimal threshold for diagnosing NAFLD.^[21]^

### 2.3. Covariates

Based on clinical experience and current literature,^[22–25]^ our multivariable models included the following potential confounding variables: sex (male, female), age (years), race (non-Hispanic White, non-Hispanic Black, Mexican American, other), education level (less than high school, high school, university and above), marital status (married/living with partner, never married/other), and income-to-poverty ratio categorized as low (≤1.3), medium (>1.3–3.5), and high (>3.5). The study assessed various health indicators, including moderate work activity, hypertension, diabetes, and smoking history (smoked at least 100 cigarettes “yes” or “no”), along with body mass index (BMI in kg/m²), glycosylated hemoglobin type A1C (HbA1c in %), albumin (g/dL), aspartate transaminase (AST, U/L), alanine transaminase (ALT, U/L), gamma glutamyl transferase (GGT, IU/L), and LSM (kPa). In subgroup analyses, age was divided into <40, 40 to 60, and ≥60 years, while BMI was categorized as <25, 25 to 30, and ≥30 kg/m².

### 2.4. Statistical analysis

With missingness ≤7.7% for every covariate, a single multivariate imputation was judged sufficient to maintain statistical power without materially distorting the estimates; multiple imputation was therefore omitted. Missing values were imputed via an iterative, round-robin algorithm employing a Bayesian Ridge estimator at each step.^[26]^ For normally distributed continuous variables, data are presented as mean ± standard deviation. For non-normally distributed continuous variables, data are shown as interquartile range. Categorical variables are expressed as percentages (%). One-way analysis of variance was employed for normally distributed data, whereas the Kruskal–Wallis test was used for non-normally distributed data. Multivariable linear regression analyses were conducted to explore the independent association between the CTI and CAP, adjusting for potential confounders. The results are expressed as beta (β) values with 95% confidence interval [CI]. The relationship between the CTI and CAP was analyzed using 3 linear regression models. Model 1 did not include covariate adjustments. Model 2 was adjusted for sex, age, and race based on previous findings and clinical constraints. Model 3, the fully adjusted model, was expanded from Model 2 by additionally adjusting for educational level, marital status, income-to-poverty ratio, moderate work activity, hypertension, diabetes, smoking history (at least 100 cigarettes), BMI, HbA1c, Albumin, ALT, AST, GGT, and LSM. In the final model, we adjusted the factors basing the following 3 rules (1 or 2 or 3): We adjusted for variables, if it was added to this model, the matched odds ratio would change by at least 10%. For univariate analysis, we adjusted for variables, of which the *P*-values < .1. 3. For multivariable analysis, variables were chosen based on previous findings and clinical constraints. Simultaneous smoothed curve fitting was achieved by adjusting for variables. A subgroup analysis examined the relationship between CTI and CAP across various demographics and health factors, including sex, age groups (<40, 40–60, ≥60), education level, marital status, income-to-poverty ratio, moderate work activity, hypertension, diabetes, smoking history (≥100 cigarettes), and BMI categories (<25, 25–30, ≥30). Statistical analyses were conducted using the Free Statistics platform (version 2.0, Beijing, China) and R software (Version 4.2.2, The R Foundation), both of which supported reproducible analyses and interactive computing. Statistical significance was defined as a 2-tailed *P*-value < .05.

## 3. Results

### 3.1. Baseline characteristics of participants

Table 1 shows the demographic, comorbidity, socioeconomic, and laboratory data of the 3248 participants. The cohort comprised 1474 males (45.4%) and 1774 females (54.6%) with a median age of 49 years. Participants were stratified into 4 groups according to CTI quartiles: Q1 (5.847–8.120), Q2 (8.120–8.730), Q3 (8.730–9.383), and Q4 (9.383–12.572).

**Table 1:** Baseline characteristics of the study population based on the c-reactive protein-triglyceride glucose index.

Variables	Total (5.847–12.572, n = 3248)	Q1 (5.847–8.120, n = 812)	Q2 (8.120–8.729, n = 812)	Q3 (8.730–9.383, n = 812)	Q4 (9.384–12.572, n = 812)	*P*-value
Sex, n (%)						.69
Male	1474 (45.4)	360 (44.3)	376 (46.3)	378 (46.6)	360 (44.3)	
Female	1774 (54.6)	452 (55.7)	436 (53.7)	434 (53.4)	452 (55.7)	
Age (yr)	48.6 ± 18.3	41.1 ± 18.7	49.2 ± 18.4	51.1 ± 17.7	53.2 ± 16.0	<.001
Race, n (%)						<.001
Non-Hispanic White	1075 (33.1)	267 (32.9)	252 (31)	262 (32.3)	294 (36.2)	
Non-Hispanic Black	791 (24.4)	246 (30.3)	209 (25.7)	197 (24.3)	139 (17.1)	
Mexican American	443 (13.6)	75 (9.2)	117 (14.4)	100 (12.3)	151 (18.6)	
Other	939 (28.9)	224 (27.6)	234 (28.8)	253 (31.2)	228 (28.1)	
Education level, n (%)						<.001
Less than high school	258 (7.9)	34 (4.2)	61 (7.5)	71 (8.7)	92 (11.3)	
High school	1055 (32.5)	234 (28.8)	252 (31)	262 (32.3)	307 (37.8)	
University and above	1935 (59.6)	544 (67)	499 (61.5)	479 (59)	413 (50.9)	
Marital status, n (%)						.006
Married/living with partner	1939 (59.7)	443 (54.6)	501 (61.7)	504 (62.1)	491 (60.5)	
Never married/other	1309 (40.3)	369 (45.4)	311 (38.3)	308 (37.9)	321 (39.5)	
Income-to-poverty ratio, n (%)						<.001
Low income	888 (27.3)	215 (26.5)	196 (24.1)	213 (26.2)	264 (32.5)	
Medium income	1263 (38.9)	283 (34.9)	331 (40.8)	324 (39.9)	325 (40)	
High income	1097 (33.8)	314 (38.7)	285 (35.1)	275 (33.9)	223 (27.5)	
Moderate work activity, n (%)						.26
Yes	1395 (42.9)	372 (45.8)	340 (41.9)	335 (41.3)	348 (42.9)	
No	1853 (57.1)	440 (54.2)	472 (58.1)	477 (58.7)	464 (57.1)	
Hypertension, n (%)						<.001
Yes	1151 (35.4)	150 (18.5)	269 (33.1)	326 (40.1)	406 (50)	
No	2097 (64.6)	662 (81.5)	543 (66.9)	486 (59.9)	406 (50)	
Diabetes, n (%)						<.001
Yes	502 (15.5)	33 (4.1)	78 (9.6)	114 (14)	277 (34.1)	
No	2746 (84.5)	779 (95.9)	734 (90.4)	698 (86)	535 (65.9)	
Smoking history, n (%)						<.001
Yes	1159 (35.7)	241 (29.7)	262 (32.3)	303 (37.3)	353 (43.5)	
No	2089 (64.3)	571 (70.3)	550 (67.7)	509 (62.7)	459 (56.5)	
BMI (kg/m^2^)	29.8 ± 7.6	25.0 ± 4.9	28.5 ± 6.1	31.4 ± 7.5	34.1 ± 8.2	<.001
HbA1c (%)	5.8 ± 1.1	5.4 ± 0.5	5.6 ± 0.6	5.8 ± 0.7	6.6 ± 1.8	<.001
Albumin (g/dL)	5.8 ± 1.1	5.4 ± 0.5	5.6 ± 0.6	5.8 ± 0.7	6.6 ± 1.8	<.001
ALT (U/L)	17.0 (12.0, 25.0)	14.0 (11.0, 19.0)	17.0 (12.0, 23.0)	19.0 (13.0, 27.0)	20.0 (14.0, 30.0)	<.001
AST (U/L)	19.0 (15.0, 23.0)	18.0 (15.0, 22.0)	19.0 (16.0, 23.0)	19.0 (16.0, 24.0)	19.0 (15.0, 25.0)	.005
GGT (IU/L)	20.0 (14.0, 30.0)	15.0 (11.0, 20.0)	19.0 (14.0, 28.0)	22.0 (16.0, 32.0)	27.0 (20.0, 43.0)	<.001
LSM (kPa)	4.9 (4.1, 6.1)	4.6 (3.9, 5.5)	4.7 (3.9, 5.8)	5.0 (4.1, 6.2)	5.5 (4.5, 7.1)	<.001
CAP (dB/m)	263.2 ± 62.8	221.9 ± 49.4	251.2 ± 54.4	275.3 ± 57.5	304.6 ± 58.3	<.001

Mean ± SD for continuous variables: the *P*-value was calculated by the linear regression model.

Median [IQR] for skewed continuous variables.

% for categorical variables: the *P*-value was calculated by the chi-square test.

ALT = alanine aminotransferase, AST = aspartate aminotransferase, BMI = body mass index, CAP = controlled attenuation parameter, GGT = gamma glutamyl transferase, HbA1c = glycosylated hemoglobin type A1C, IQR = interquartile range, LSM = liver stiffness measure, Q = quartile, SD = standard deviation.

Significant differences were observed among the quartiles in the distributions of age, race, education level, marital status, income-to-poverty ratio, hypertension, diabetes, and smoking history. Participants in Q4 had higher BMI, HbA1c, albumin, ALT, AST, GGT, and LSM levels. Participants in Q4 demonstrated higher CAP values.

### 3.2. Association between CTI and CAP

**Figure 2.**
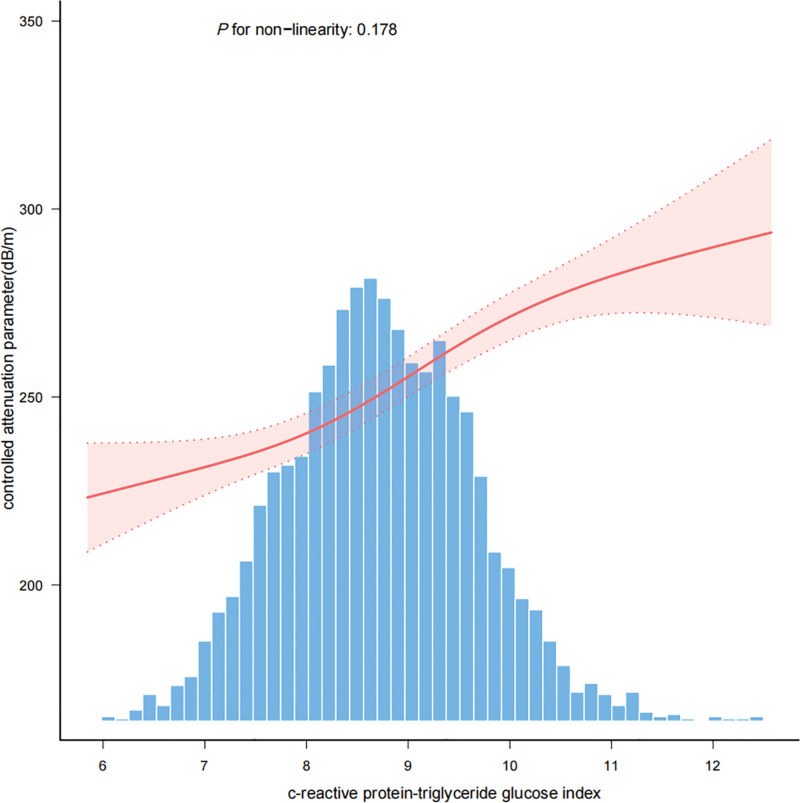
The association between c-reactive protein-triglyceride glucose index and controlled attenuation parameter. Smooth curve fitted by a generalized additive model. The solid red line denoted the fitted curve and the 2 dashed red lines represented 95% confidence intervals. Model adjusted for sex, age, race, education level, marital status, income-to-poverty ratio, moderate work activity, hypertension, diabetes, smoking history, BMI, HbA1c, ALT, albumin, AST, GGT, and LSM. ALT = alanine aminotransferase, AST = aspartate aminotransferase, BMI = body mass index, GGT = gamma glutamyl transferase, HbA1c = glycosylated hemoglobin, type A1C, LSM = liver stiffness measure.

Three linear regression models were employed to examine the association between the CTI and CAP. All 3 models demonstrated a positive association between CTI and CAP: Model 1 (β = 33.79; 95% CI: 31.8–35.78), Model 2 (β = 31.80; 95% CI: 29.75–33.86), and Model 3 (β = 13.85; 95% CI: 11.51–16.2) (Table 2). The CTI was stratified into quartiles, and trend testing across all 3 models revealed a significant linear relationship between the CTI and CAP (*P* < .001) (Table 2). Smooth curve fitting was employed to explore the potential nonlinear relationships, ultimately confirming a positive linear relationship (*P* for nonlinearity = .178) as shown in Figure 2.

**Table 2:** Multivariable linear regression analysis of c-reactive protein-triglyceride glucose index and controlled attenuation parameter.

Variable	N	Model 1		Model 2		Model 3	
		β (95% CI)	*P*-value	β (95% CI)	*P*-value	β (95% CI)	*P*-value
CTI	3248	33.79 (31.8, 35.78)	<.001	31.80 (29.75, 33.86)	<.001	13.85 (11.51, 16.2)	<.001
Quartiles of CTI							
Q1	812	0 (Ref)		0 (Ref)		0 (Ref)	
Q2	812	29.33 (23.98, 34.68)	<.001	25.64 (20.28, 30.99)	<.001	9.01 (4.18, 13.84)	<.001
Q3	812	53.4 (48.05, 58.75)	<.001	49.17 (43.78, 54.56)	<.001	19.52 (14.4, 24.64)	<.001
Q4	812	82.75 (77.4, 88.1)	<.001	76.93 (71.45, 82.42)	<.001	31.32 (25.51, 37.14)	<.001
*P* for trend			<.001		<.001		<.001

Model 1 unadjusted.

Model 2 adjusted for sex, age, and race.

Model 3 was further adjusted for education level, marital status, income-to-poverty ratio, moderate work activity, hypertension, diabetes, smoking history, BMI, HbA1c, ALT, albumin, AST, GGT, and LSM based on Model 2.

ALT = alanine aminotransferase, AST = asparate aminotransferase, BMI = body mass index, CI = confidence interval, CTI = c-reactive protein-triglyceride glucose index, GGT = gamma glutamyl transferase, HbA1c = glycosylated hemoglobin type A1C, LSM = liver stiffness measure, Q = quartiles, Ref = reference, β = beta.

### 3.3. Subgroup analyses

**Figure 3.**
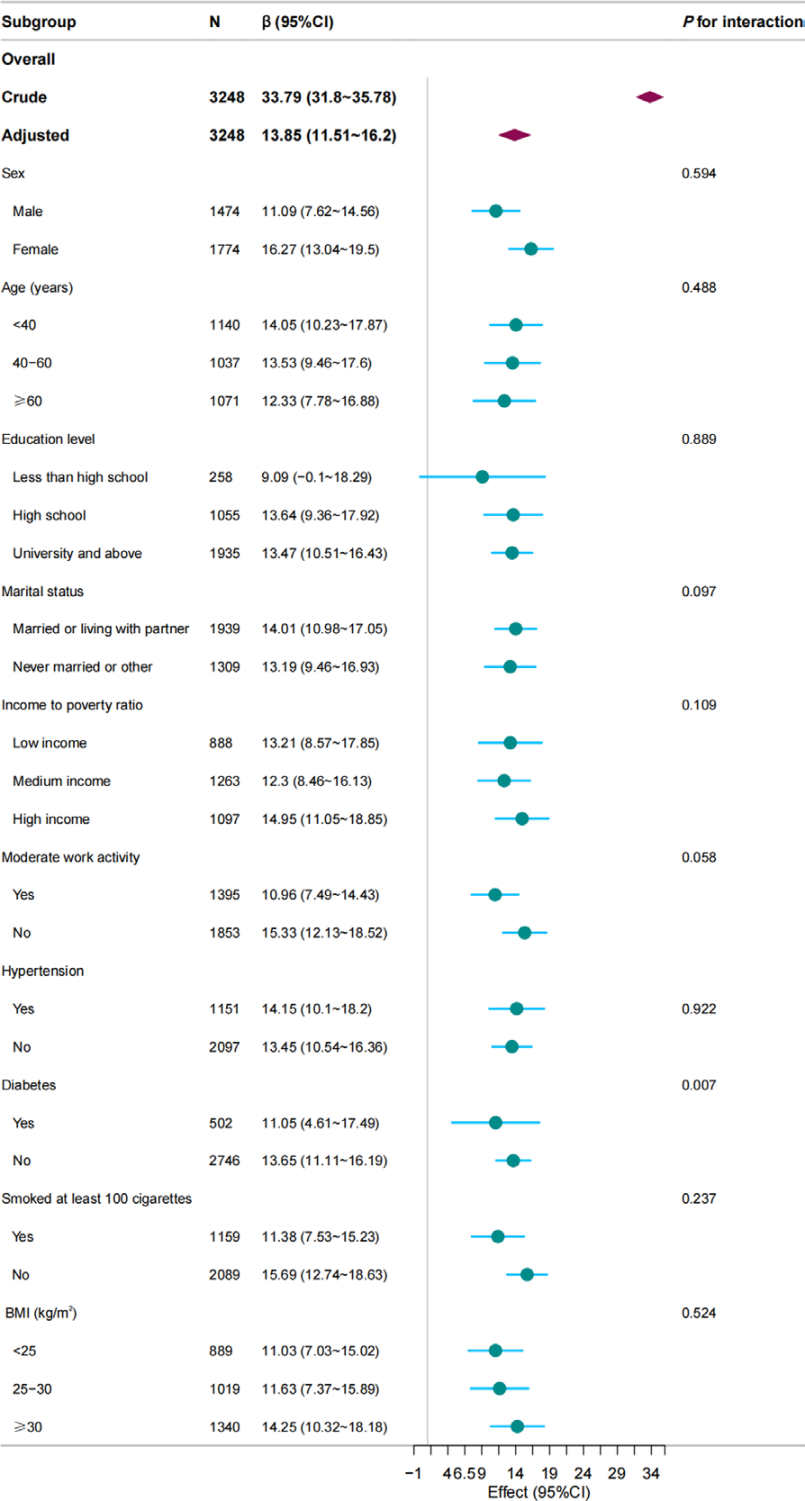
Subgroup analysis for the association between c-reactive protein-triglyceride glucose index and controlled attenuation parameter. Covariates to be adjusted included sex, age, race, education level, marital status, hypertension, income-to-poverty ratio, moderate work activity, hypertension, diabetes, smoking history, BMI, HbA1c, ALT, Albumin, AST, GGT, and LSM and covariates related to stratification factors were not adjusted. ALT = alanine aminotransferase, AST = asparate aminotransferase, BMI = body mass index, CI = confidence interval, GGT = gamma glutamyl transferase, HbA1c = glycosylated hemoglobin type A1C, LSM = liver stiffness measure, β = beta.

Participants were grouped into distinct subgroups based on sex, age, education level, marital status, income-to-poverty ratio, moderate work activity, hypertension, diabetes, smoking history, and BMI. Following the adjustment for confounders, the β coefficients of CTI on CAP demonstrated consistency across subgroups (all β > 0). Despite *P* < .05 for the interaction of diabetes, the findings may not be clinically significant considering the similar directionality of the associations. The details are shown in Figure 3.

## 4. Discussion

Timely identification of NAFLD is critical for providing opportunities to implement interventions capable of altering its natural progression.^[27]^ This cross-sectional study included 3248 adults from the United States. In the comprehensive regression model accounting for all covariates, a positive correlation was identified between the combined CTI and CAP. A smooth curve analysis revealed a linear relationship. The consistent association between the CTI and CAP was further substantiated by subgroup analyses and interactions.

CTI, a novel indicator that provides a comprehensive evaluation of inflammation and IR, is potentially valuable for predicting survival in patients with cancer.^[19,20]^ However, the exact mechanism of the close correlation between the CTI and NAFLD has not been fully elucidated, and it may be related to IR, inflammation, endothelial dysfunction, dysregulated glycolipid metabolism, and thrombosis.^[28]^ Previous studies have shown a nonlinear relationship between the triglyceride glucose index and all-cause mortality.^[29]^ Insulin is crucial for maintaining the balance between contractile and diastolic functions of vascular endothelial cells under normal physiological conditions. This regulation occurs through 2 primary signaling pathways: phosphatidylinositol 3-kinase- and mitogen-activated protein kinase-dependent signaling. However, this delicate balance is disrupted in the presence of IR, leading to endothelial dysfunction and subsequent glycolipotoxicity throughout the organism. Excessive fat accumulation in the liver stimulates ROS production within the mitochondria. This results in a significant increase in oxidized low-density lipoproteins circulating in the bloodstream, which in turn triggers endothelial oxidative stress injury and impairs vascular function.^[30,31]^ A retrospective study in Korea involving participants aged 10 to 19 years found that the triglyceride glucose index was strongly associated with NAFLD in this population, especially in females.^[32]^

Our findings further support the association between the CTI and NAFLD. In a study of 6558 participants, the hs-CRP level was elevated in NAFLD and was independently associated after adjustment.^[33]^ As reported by Cui et al, CRP has been shown to have a significant mediating role in the association between the triglyceride glucose index and cardiovascular events.^[34]^ Pursuing a regular physical exercise regimen, implementing dietary modifications, maintaining an optimal weight, and employing judicious pharmacological therapies to mitigate IR and inflammation may reduce the development of NAFLD. In addition, we propose that evaluating inflammation and IR together may have prognostic significance for cardiovascular events in patients with NAFLD. Further studies are needed to explore the association between CTI and cardiovascular disease in this population.

In this study, LSM, another key indicator of VCTE, showed a statistically significant upward trend with increasing CTI (Table 1). However, in multivariate logistic regression analysis, the correlation with LSM was unstable. Li et al^[35]^ found that the triglyceride glucose index positively correlated with CAP; however, no significant association was observed between the triglyceride glucose index and LSM. This may explain the instability of the correlation analysis of LSM in this study, indicating that there is a complex relationship between the CTI and LSM, which requires further research, highlighting the potential of the CTI as an effective tool for identifying NAFLD. The integration of inflammatory markers with IR may offer a novel approach for the diagnosis of NAFLD.

Our analysis, adjusted for various confounders, consistently showed a positive association between CTI and NAFLD, regardless of whether CTI was treated as a continuous or categorical variable. The incorporation of covariates in our analysis was informed by their recognized or hypothesized associations with the CTI and NAFLD. As previously mentioned, there is a robust correlation between CRP and the triglyceride glucose index, both of which have been demonstrated to be significantly associated with NAFLD. Consequently, there is a possibility of a correlation between the CTI and NAFLD, a hypothesis substantiated by this study’s findings. The CTI is a simple and cost-effective screening tool that can be effectively implemented in primary care settings to identify individuals with an increased likelihood of developing NAFLD, and demonstrates potential value in optimizing diagnostic models.

This study has several limitations. First, as a cross-sectional study, our findings reflect associations between variables rather than causal relationships. Therefore, we cannot establish a causal link between CTI and NAFLD. Second, this study utilized CAP to assess the severity of hepatic steatosis. However, this method has certain limitations: it cannot accurately differentiate between adjacent grades of steatosis, and its diagnostic accuracy is susceptible to factors such as obesity, elevated ALT levels, and operator dependence. Therefore, further validation using biopsy-confirmed cohorts remains necessary for the findings of this study. Finally, although the NHANES was conducted in a diverse adult population in the United States, the findings may not apply to other regions or ethnic groups.

## 5. Conclusion

The results of this study showed a positive association between the CTI and CAP in adults in the United States, suggesting its potential as a reliable indicator for NAFLD monitoring.

## Acknowledgments

We thank the staff at the National Center for Health Statistics of the Centers for Disease Control and Prevention for designing, collecting, and collating the NHANES data and creating a public database.

## Author contributions

**Conceptualization:** Yuanyuan Chen, Bing Yang, Jun Chen, Chuantie Chen.

**Data curation:** Jinmin Cao, Huihui Chen.

**Formal analysis:** Bing Yang, Jun Chen.

**Funding acquisition:** Yuanyuan Chen, Chuantie Chen.

**Methodology:** Huihui Chen, Chuantie Chen.

**Resources:** Jinmin Cao.

**Software:** Yuanyuan Chen, Chuantie Chen.

**Supervision:** Bing Yang, Jun Chen.

**Writing – original draft:** Yuanyuan Chen.

**Writing – review & editing:** Yuanyuan Chen, Chuantie Chen.
